# Preclinical Evaluation of Liposomal C8 Ceramide as a Potent anti-Hepatocellular Carcinoma Agent

**DOI:** 10.1371/journal.pone.0145195

**Published:** 2016-01-04

**Authors:** Huiqing Lv, Zhongmin Zhang, Xiaoyu Wu, Yaoxia Wang, Chenglin Li, Weihong Gong, Liang Gui, Xin Wang

**Affiliations:** 1 Department of Hyperbaric Oxygen, Lin Yi People's Hospital, Lin Yi, China; 2 Department of Oncology, Lin Yi People's Hospital, Lin Yi, China; 3 Department of General Surgery, The Affiliated Hospital of Nanjing Medical University. Nanjing, China; University of Medicine, Greifswald, Germany, GERMANY

## Abstract

Hepatocellular carcinoma (HCC) remains a global health threat. The search for novel anti-HCC agents is urgent. In the current study, we synthesized a liposomal C8 ceramide, and analyzed its anti-tumor activity in pre-clinical HCC models. The liposomal C8 (ceramide) potently inhibited HCC cell (HepG2, SMMC-7721 and Huh-7 lines) survival and proliferation, more efficiently than free C8 ceramide. Yet, non-cancerous HL7702 human hepatocytes were resistant to the liposomal C8 treatment. Liposomal C8 activated caspase-dependent apoptosis in HCC cells, and HCC cytotoxicity by liposomal C8 was significantly attenuated with co-treatment of caspase inhibitors. At the molecular level, we showed that liposomal C8 activated ASK1 (apoptosis signal-regulating kinase 1)-JNK (Jun N-terminal protein kinase) signaling in HCC cells. On the other hand, JNK pharmacological inhibition or dominant negative mutation, as well as ASK1 shRNA-knockdown remarkably inhibited liposomal C8-induced apoptosis in HCC cells. Further studies showed that liposomal C8 inhibited AKT-mTOR (mammalian target of rapamycin) activation in HCC cells. Restoring AKT-mTOR activation by introducing a constitutively-active AKT alleviated HepG2 cytotoxicity by liposomal C8. *In vivo*, intravenous (*i*.*v*.) injection of liposomal C8 significantly inhibited HepG2 xenograft growth in severe combined immuno-deficient (SCID) mice, and mice survival was significantly improved. These preclinical results suggest that liposomal C8 could be further studied as a valuable anti-HCC agent.

## Introduction

Hepatocellular carcinoma (HCC) is a global health threat [1,2,3]. It is the third leading cause of cancer-related mortality, causing over 600,000 death annually [1,2,3,4]. Its incidence, however, has been steadily increasing at an alarming rate, and over half a million people will be diagnosed with this devastating disease each year [4]. Due to the lack of specific clinical symptoms, the fast majority of HCCs were diagnosed at late and/or advanced stages [2]. HCC ranks one of the most resistance tumors to possible all conventional chemotherapeutic agents [5,6]. Therefore, there is an urgent need to explore novel and more efficient chemotherapeutic/chemo-preventive agents for HCC [5,6].

Ceramides, a family of lipid molecules composed of sphingosine and fatty acid, are found in high concentrations within cell membranes [7,8]. Ceramides could also act as active signaling molecules regulating several key cellular functions [7,8]. Existing evidences have established a link between ceramide level and cell apoptosis, and proposed that processes that could facilitate intracellular ceramide accumulation will produce a pro-apoptotic outcome [7,8,9]. As a matter of fact, a number of chemotherapeutic agents can induce intracellular ceramide production to promote cell apoptosis [7,8].

The cell-permeable short-chain ceramides (C2, C4, C6 and C8) have displayed significant anti-tumor activity against a variety of cancer cells (reviewed in [10]). These short-chain ceramides exerted potent anti-survival, anti-proliferative and pro-apoptotic activities against different cancer cells [11]. The use of these short-chain ceramides is severely limited due to their extremely poor solubility [12]. Therefore, liposome-based nanotechnology drug delivery systems have been developed to assist short-chain ceramide delivery [12].

Recent studies have shown that C6 ceramide and liposomal C6 ceramide could significantly inhibit HCC cell growth [13,14,15]. In the current study, we were able to synthesize a liposomal C8 (ceramide). This study analyzed the potential role of the liposomal C8 against human HCC cells both *in vitro* and *in vivo*. The underlying signaling mechanisms were also studied.

## Material and Methods

### 2.1. Cell culture

Human HCC cell lines, HepG2, SMMC-7721 and Huh-7, were purchased from the Cell Bank of Shanghai Biological Institute CAS (Shanghai, China). HCC cells were maintained in DMEM/RPMI-1640 medium supplemented with 5% fetal bovine serum (FBS) at 37°C. The human HL-7702 hepatocytes were obtained from the Fudan University IBS center (Shanghai, China), and were cultured in RPMI-1640 containing 10% FBS. Cells were changed to serum-free medium 24 h before the assay. All cell culture reagents were obtained from Gibco Life Technologies (Shanghai, China).

### 2.2. Liposomal C8 ceramide preparation

Ceramide liposomes and control liposomes (no C8 ceramide) were prepared, briefly, lipids were solubilized in chloroform, combined in a specific molar ratio DSPC/1,2-dioleoyl-*sn*-glycero-3-phosphoethanolamine/1,2-distearoyl-*sn*-glycero-3-phosphoethanolamine-*N*-[methoxyPEG(2000)]/*N*-octanoyl-sphingosine-1-[succinyl(methoxyPEG(750))]/C8-ceramide (4.9:2.6:1.2:1:3 M ratio), dried under nitrogen, and hydrated with sterile isotonic 0.9% NaCl solution. Liposomes were prepared by sonication and extrusion of the solution through 100-nm polycarbonate membranes. Ceramide loading in liposomes was 14% (w/w). The liposomes were kept at 4°C in saline at a concentration of 25 mg/mL (containing 3.5 mg/mL C8 ceramide) before use. All lipids were purchased from Avanti (Alabaster, AB).

### 2.3. Chemicals and reagents

The caspase-3 inhibitor Z-VAD-fmk and the caspase-9 inhibitor Z-LEHD-fmk were purchased from Calbiochem (Shanghai, China). The two JNK (Jun N-terminal protein kinase) inhibitors, SP600125 and JNK inhibitor IX (JNKi-IX), were purchased from Selleck (Shanghai, China). p-AKT (Ser 473) antibody (9271), AKT1 antibody (2938), p-S6 ribosomal protein (S6, Ser 235/236) antibody (2211), S6 antibody (2317), p-p70 S6 kinase 1 (S6K1, Thr 398) antibody (9209), S6K1 antibody (9202), p-SAPK/JNK1 (Thr183/Tyr185) antibody (9251), Jun N-terminal protein kinase (JNK1) antibody (3708), p-ASK1 (Thr 845) antibody (3765), ASK1 antibody (3762), cyclin D1 antibody (2922), HIF-1α antibody (3716), c-Jun antibody (9165) and (β-)Tubulin (D2N5G) antibody (15115), cleaved-caspase-3 antibody (9661) and β-actin antibody (3700) were all purchased from Cell Signaling Tech.(Beverly, MA).

### 2.4. MTT cell proliferation assay

Cell proliferation was analyzed by 3-(4,5-dimethylthiazol-2-yl)-2,5-diphenyl tetrazolium bromide (MTT, Sigma) assay. Briefly, cells (3 × 10^3^ cells/well) were seeded onto the 96-well tissue culture plate. Following indicated treatments, twenty μL of MTT (5 mg/mL) was added to the wells, and incubated for additional 2 h. Absorbance was recorded at 590 nm using an Eliza Plate Reader.

### 2.5. Clonogenicity assay

As previously reported [16], one thousand cells per dish were trypsinized and suspended in 1 mL complete medium containing 0.5% low-melting-point agarose (Sigma). The agar-cell mixture was then plated on top of a bottom layer with 0.5% complete medium agar mixture. Two weeks following indicated treatment, colonies were stained with 0.1% crystal violet, and viable colonies were manually counted.

### 2.6. Cell death assay

Following indicated treatment, cell death ratio was calculated by counting the cells in a Neubauer chamber. An aliquot of the total cell suspension (2 × 10^5^ cells) was mixed with an equal volume of trypan blue, and incubated for 5 min at room temperature. Cell death ratio was calculated by the number of trypan blue positive cells divided by the total cell number × 100%.

### 2.7. Quantification of apoptosis by ELISA

As previously reported [17], the Histone DNA Cell Apoptosis ELISA Detection Kit (Roche, Palo Alto, CA) was utilized to quantify cell apoptosis according to the manufacturer's protocol.

### 2.8. Caspase-3/-9 activity assay

Cells were lysed in the buffer containing 5 mM Tris (pH 8), 20 mM EDTA, and 0.5% Triton-X100. Reaction mixture contained 20 mM HEPES (pH 7), 10% glycerol, 2 mM dithiothreitol, 30 *μ*g protein per condition, and 20 *μ*M of the caspase-3 substrate (Ac-DEVD-AFC), or caspase-9 substrate (Ac-LEHD-AFC). Enzymatic activity was measured at excitation wavelength of 380 nm and emission wavelength of 440 nm.

### 2.9. TUNEL staining assay

Following applied treatment, TUNEL (Terminal deoxynucleotidyl transferase dUTP nick end labeling) In Situ Cell Death Detection Kit (Roche) was utilized to test cell apoptosis according to the manufacturer’s instructions. Cells were also stained with 4’,6’-diamino-2-phenylin-dole (DAPI, Roche) to visualize cell nuclei. Cell apoptosis percentage was calculated by the TUNEL percentage (TUNEL vs. DAPI) detected under a fluorescence microscope (Zeiss). At least 500 cells in 10 random views (1: 200 magnification) for each condition were included to calculate TUNEL percentage. Representative images were also shown.

### 2.10. Apoptosis assay by PI-annexin V staining

After applied treatment, the floating and attached cells were collected. Cells were then washed with cold PBS, and incubated with 0.5 mL of binding buffer (10 mM HEPES, pH 7.4, 150 mM NaCl, 2.5 mM CaCl_2_, 1 mM MgCl_2_, and 4% BSA), with 5.0 μg/mL Annexin V-FITC (Invitrogen) for 15 min under dark. Cells were also washed with PBS and resuspended in binding buffer with 2.0 μg/mL propidium iodide (PI) (Invitrogen). A total of 10,000 cells per sample were analyzed by flow cytometry in a FACS (BD, Shanghai, China). Annexin V percentage was recorded as a quantitative apoptosis measurement.

### 2.11. Western blots

After applied treatment, the equal amount of protein samples (30 μg/sample) were separated by electrophoresis in SDS-PAGE gels. Proteins were then transferred to the PVDF membranes, and detected with the specific primary antibodies. The immuno-reactive proteins after incubation with corresponding secondary antibodies were detected with the enhanced chemiluminescence (ECL) system (Amersham, Buckinghamshire). Band intensity was quantified through ImageJ software, and was normalized to the corresponding loading control. All the blot data in this paper were repeated four times, and similar results were obtained.

### 2.12. ASK1 (apoptosis signal-regulating kinase 1) shRNA knockdown and stable cell selection

HepG2 cells were seeded onto the six-well plate with 50% confluence plus polybrene (Sigma). ASK1-shRNA (h) lentiviral particles (sc-29748-V, Santa Cruz Biotech, Santa Cruz, CA) (10 μL/mL medium) were directly added to cultured HepG2 cells for 12 h. Cells were then cultured in fresh medium for additional 24 h. Control cells were incubated with same amount of scramble shRNA lentiviral particles (sc-108080-V, Santa Cruz) (10 μL/mL medium). Stably cells were selected by culturing in the presence of puromycin (2.5 μg/ml) for 10–12 days. ASK1 expression in stable cells was verified by Western blots.

### 2.13. Dominant negative JNK1 construct and stable cell line establishment

The dominant negative JNK1 (dn-JNK1), achieved by switching Lys in the ATP-binding domain to Arg, was designed and synthesized by Genechem (Shanghai, China). The cDNA was inserted into pSuper-puro plasmid (a gift from Dr. Dongfeng Xu’s Lab). The dn-JNK1 plasmid or the empty vector (pSuper-puro) was then transfected into HEK-293 cells together with viral packaging protein plasmids VSVG and Hit-60 (Promega) with the Lipofectamine 2000 (Invitrogen) reagent. The virus-containing supernatants were collected and filtered, and were then added to HepG2 cells (seeded onto the six-well plate with 50% confluence with polybrene). Stably cells were selected by culturing in the presence of puromycin (2.5 μg/ml) for 10–12 days. JNK1 expression in stable cells was verified by Western blots.

### 2.14. Constitutively active-AKT1 (ca-AKT1) transfection and stable cell line selection

The adenoviral vector expressing a constitutively active AKT1 (ca-AKT1) and the empty vector expressing green fluorescence protein (Ad-GFP) were gifts from Dr. Teng [18], and were verified in our lab. ca-AKT1 or the empty vector (Ad-GFP) (0.25 μg/mL) was transfected into HepG2 cells (seeded onto the six-well plate with 50% confluence) using the Lipofectamine 2000 protocol. Stably cells were selected by culturing in the presence of puromycin (2.5 μg/ml) for 10–12 days. After selection, more than 90% of cells were GFP positive. Western blots were utilized to test AKT expression in stable cells.

### 2.15. Tumor xenografts

The male severe combined immuno-deficient (SCID) mice were implanted subcutaneously (*s*.*c*.) with a significant amount of HepG2 cells (1×10^7^ per mouse). After 7–10 days, the tumors reached the average volume of 100 mm^3^, the mice were divided into four groups: Saline, low-dose of liposomal C8 ceramide (5 mg C8 ceramide/5 mL saline/kg), high-dose of liposomal C8 ceramide (15 mg C8 ceramide/5 mL saline/kg), the empty liposomes (no ceramide), with 10 mice per group. Saline or liposomes were injected intravenously (*i*.*v*.) every 2 days, for a total of 30 days. The tumor volumes were recorded every 10 days, calculated using formula: π/6 × larger diameter × (smaller diameter)^2^. For the mice survival studies, humane endpoints were utilized to minimize mice suffering. Animals were observed every day. Clinical signs such as significant-reduced locomotion, signs of severe diarrhea, severe piloerection or a sudden weight loss (>20%) were recorded. If animals reached these endpoints they were euthanized by exsanguination under 2,2,2-tribromoethanol anesthesia (4 mg/10 g body weight, Sigma). All injections were performed under the 2,2,2-tribromoethanol anesthesia method. Animal studies have been approved by the Nanjing Medical University’s Institutional Animal Care and Use Committee (IACUC, approval number 201403128, contact person: Dr. Wu Jun).

### 2.16. Statistical analysis

Results were compared by one-way analysis of variance (ANOVA) followed by Turkey's test. All data was expressed as mean ± standard deviation (SD). A value of ***p*** < 0.05 was considered as statistically significant.

## Results

### 3.1. Liposomal C8 ceramide inhibits HCC cell proliferation and survival

**Fig 1:**
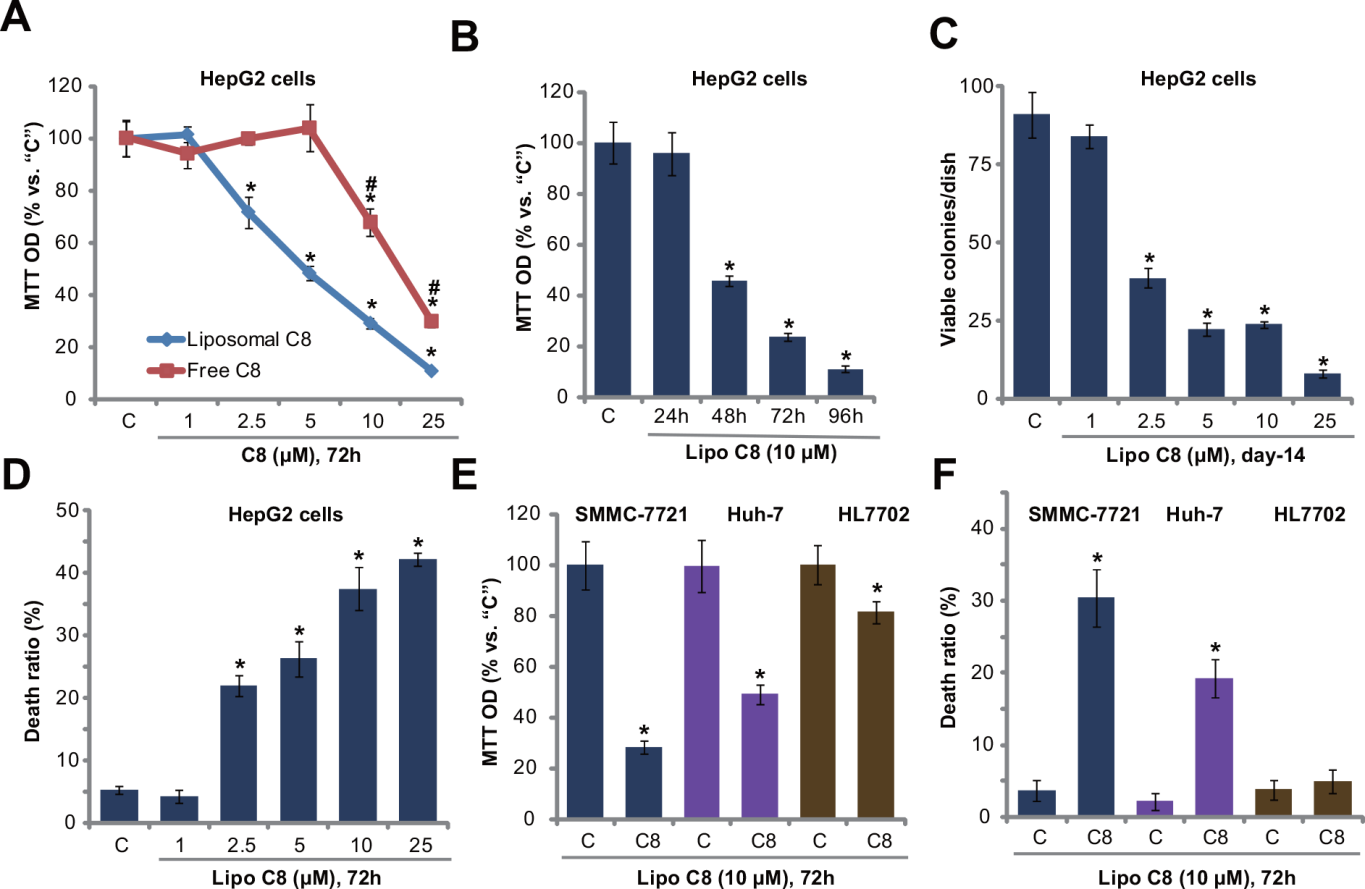
Liposomal C8 ceramide inhibits HCC cell proliferation and survival. HepG2 (A-D), SMMC-7721 (E and F) and Huh-7 (E and F) human HCC cells, as well as HL7702 human hepatocytes (E and F) were either left untreated (“C”, same for all figures), or treated with applied concentrations of liposomal C8 ceramide (“Lipo C8”, same for all figures) or free C8 ceramide (For “A”) for indicated time, cell proliferation was tested by MTT assay (A, B and E) or clonogenicity assay (C, for HepG2 cells), and cell death was tested by trypan blue staining assay (D and F). Data represent the means of three independent experiments ± standard deviations (SD). The asterisks (*) indicate statistically significant differences compared to “C” group.

First, we tested the potential effect of liposomal C8 (ceramide) on HCC cell proliferation, and compared its activity with free C8 ceramide. MTT assay results in Fig 1A demonstrated that liposomal C8 efficiently inhibited HepG2 cell proliferation in a dose-dependent manner. Its activity was significantly more potent than same free C8 ceramide (Fig 1A). For example, at 5 μM, liposomal C8 induced over 50% proliferation inhibition, while free C8 had almost no effect on HepG2 cells (Fig 1A). The anti-proliferation activity appeared also time-dependent. A remarkable proliferation inhibition was noticed 48 h after liposomal C8 (10 μM) treatment. Clonogenicity assay results in Fig 1C further confirmed the anti-HepG2 activity by liposomal C8.

At the meantime, as shown in Fig 1D, the number of trypan blue positive (“dead”) HepG2 cells was significantly increased following 2.5–25 μM of liposomal C8 treatment, indicating that liposomal C8 ceramide inhibited HepG2 cell survival. MTT assay results in Fig 1E and trypan blue staining assay results in Fig 1F showed that liposomal C8 exerted anti-proliferation and pro-death activities in two other HCC cell lines: SMMC-7721 and Huh-7. Yet, same liposomal C8 treatment induced much weaker cytotoxic effect to HL7702 human hepatocytes (non-cancerous cells), leading to less than 20% survival reduction and almost no apoptosis (Fig 1E and 1F). Free C8 at 10 μM was unable to inhibit survival of above SMMC-7721/Huh-7 cells, nor HL7702 cells (S1 Fig). Notably, the liposomal vehicle control showed no effect on HCC cell survival or proliferation (Data not shown). Therefore, these results demonstrate that liposomal C8 exerts potent anti-proliferation and pro-death activities against human HCC cells.

### 3.2. Liposomal C8 ceramide induces caspase-dependent apoptosis in HCC cells

**Fig 2:**
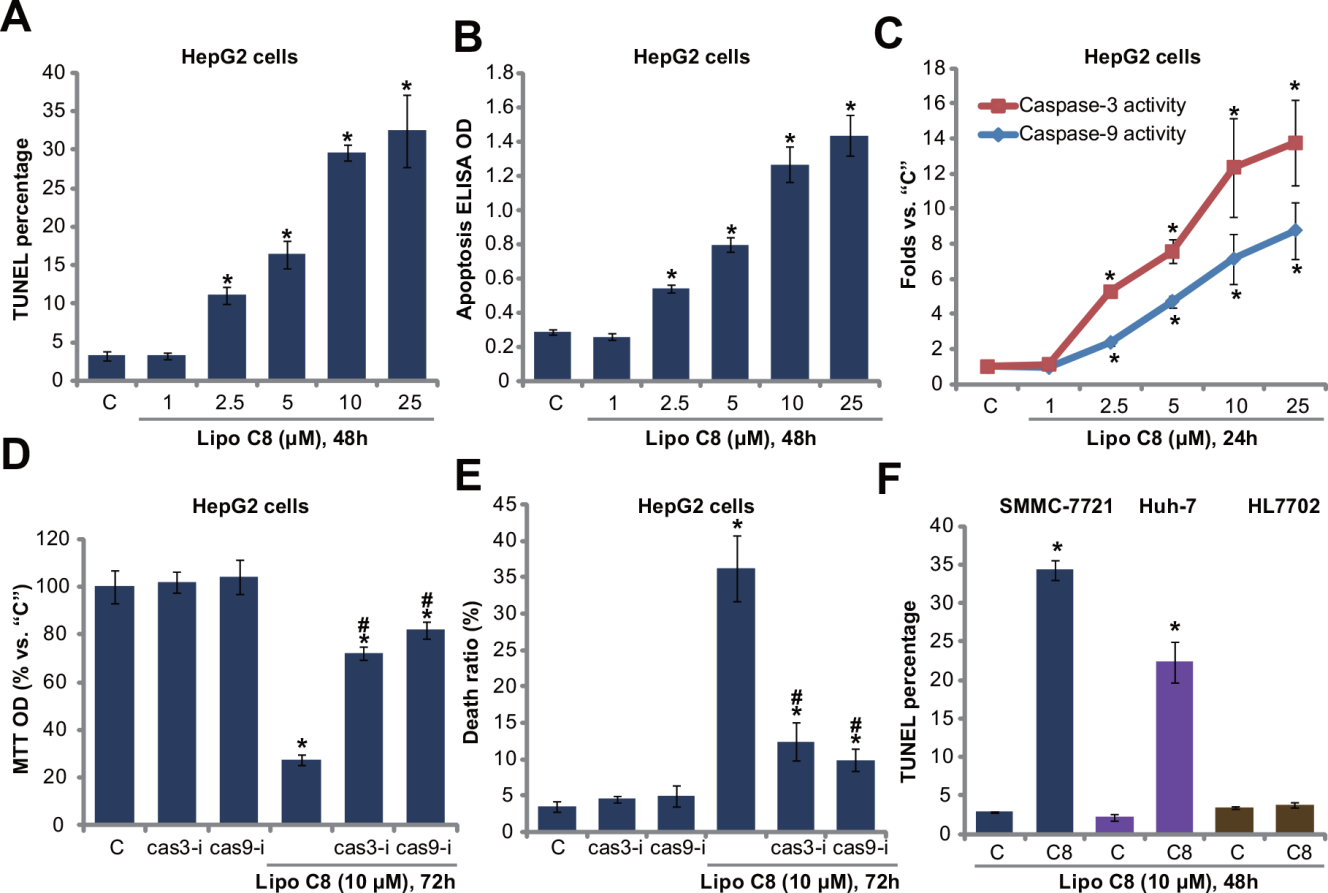
Liposomal C8 ceramide induces caspase-dependent apoptosis in HCC cells. HepG2 (A-E), SMMC-7721 (F) and Huh-7 (F) human HCC cells, as well as HL7702 human hepatocytes (F) were treated with applied concentrations of liposomal C8 for indicated time, cell apoptosis was tested by the assays described (A-C, F). HepG2 cells, pretreated with the caspase 3 specific inhibitor z-VAD-fmk (“cas3-i”, 30 μM) or the caspase-9 specific inhibitor Z-LEHD-fmk (“cas9-i”, 30 μM) for 1 h, were treated with liposomal C8 (10 μM), cells were then further cultured, and cell proliferation and cell death were tested by MTT assay (D) and trypan blue assay (E), respectively. Data represent the means of three independent experiments ± SD. The asterisks (*) indicate statistically significant differences compared to “C” group. ^#^ indicates statistically significant differences compared to liposomal C8 only group (D and E).

Next, we studied the potential role of liposomal C8 on HCC cell apoptosis. HepG2 cells were treated indicated concentrations of liposomal C8, and following cell apoptosis was tested by three independent assays: TUNEL staining assay (Fig 2A and S2 Fig), histone DNA apoptosis ELISA assay (Fig 2B) and caspase-3/-9 activity assay (Fig 2C). Results from all three assays showed that liposomal C8 induced significant apoptosis activation in HepG2 cells. The effect of liposomal C8 was once again dose-dependent (Fig 2A–2C).

To study the role of apoptosis in liposomal C8-indcued HepG2 cytotoxicity, caspase inhibitors were applied. As demonstrated, the caspase 3 specific inhibitor z-VAD-fmk and the caspase-9 specific inhibitor Z-LEHD-fmk significantly attenuated liposomal C8-indcued HepG2 cell growth inhibition (MTT OD reduction, Fig 2D) and cell death (Fig 2E), indicating that apoptosis activation was required for liposomal C8-mediated cytotoxicity against HepG2 cells. Liposomal C8-induced apoptosis activation was also observed in SMMC-7721 and Huh-7 HCC cells (Fig 2F). Yet, same liposomal C8 treatment failed to induce significant apoptosis (TUNEL increase) in HL7702 human hepatocytes (Fig 2F). Notably, the liposomal vehicle control had no significant effect on HCC or HL770 cell apoptosis. Collectively, these results demonstrate that liposomal C8 activates caspase-dependent apoptosis in HCC cells

### 3.3 ASK1-JNK activation contributes to liposomal C8 ceramide-induced activity against HCC cells

**Fig 3:**
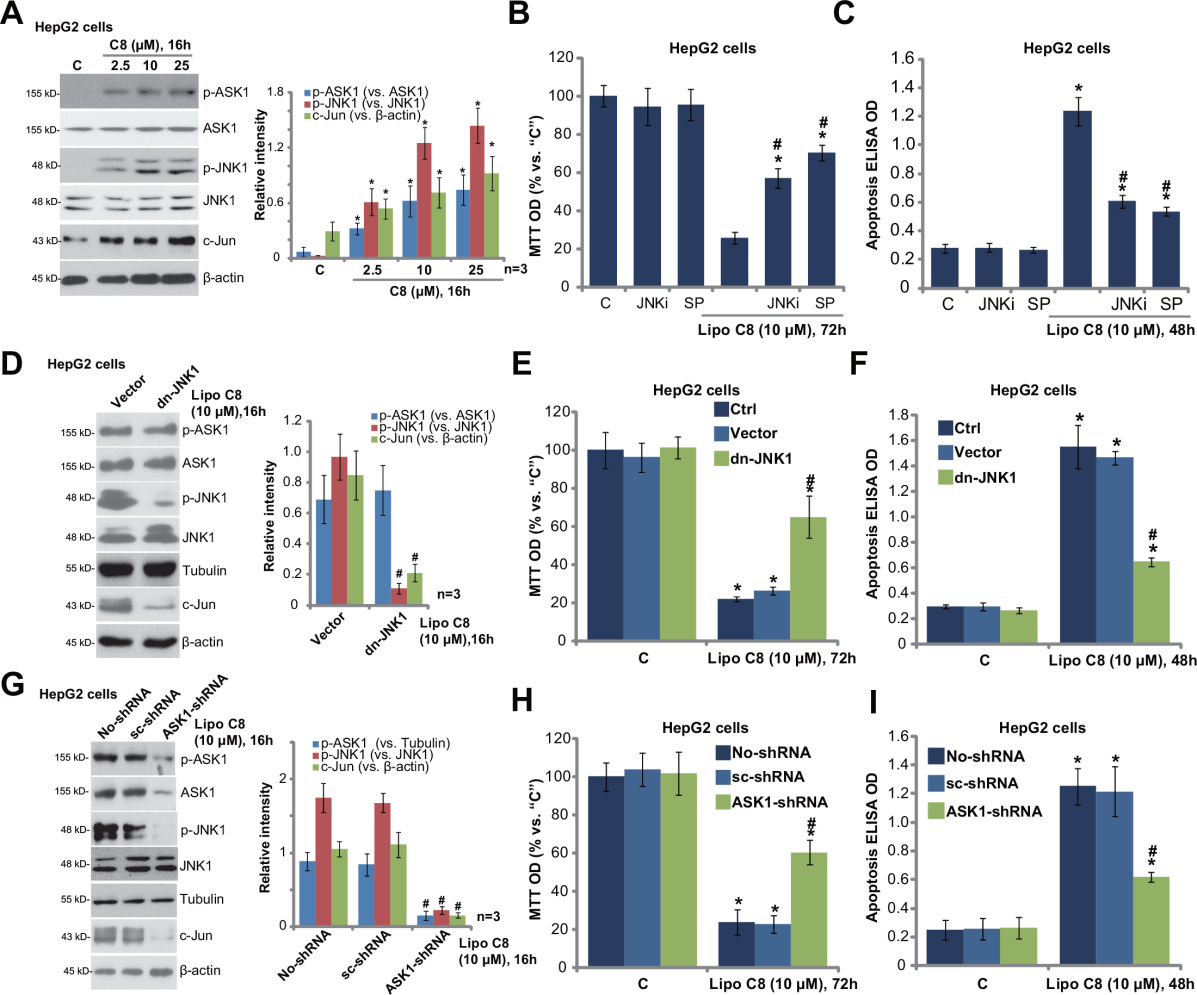
ASK1-JNK activation contributes to liposomal C8 ceramide-induced activity against HCC cells. HepG2 cells were treated with applied concentrations of liposomal C8 for indicated time, expressions of indicated proteins were tested Western blots (A). HepG2 cells, pretreated with JNK inhibitor IX (“JNKi”, 0.25 μM) or SP600125 (“SP”, 5 μM) for 1 h, were treated with liposomal C8 (10 μM), cell proliferation (B) and apoptosis (C) were tested. Control HepG2 cells, or the stable HepG2 cells expressing dominant negative JNK1 (“dn-JNK1”) or empty vector (pSuper), were treated with liposomal C8 (10 μM) for applied time, Western blots were utilized to test the signaling changes (D), cell proliferation (E) and cell apoptosis (F) were also tested. Control HepG2 cells, as well as stable HepG2 cells expressing scramble control shRNA (“sc-shRNA”) or ASK1-shRNA were treated with liposomal C8 (10 μM) for applied time, signaling changes (G), cell proliferation (H) and apoptosis (I) were tested as described. Expressions of listed proteins were quantified (A, D and G, a total of three repeats). Data represent the means of three independent experiments ± SD. The asterisks (*) indicate statistically significant differences compared to “C” group. ^#^ indicates statistically significant differences compared to liposomal C8 only group of “no shRNA” or “Vector” group.

Next, we studied the molecular changes by liposomal C8 treatment in HCC cells. Western blot results in Fig 3A demonstrated that liposomal C8 induced significant ASK1-JNK phosphorylations (activation) in HepG2 cells. c-Jun, downstream of ASK-JNK signaling axis [19,20,21], was also upregulated in liposomal C8-treated HepG2 cells (Fig 3A). Notably, the liposomal vehicle control had no significant effect on ASK1-JNK activation in HepG2 cells (Data not shown). Significantly, two JNK inhibitors, including JNK inhibitor IX and SP600125, dramatically attenuated liposomal C8-induced growth inhibition (Fig 3B) and apoptosis (Fig 3C and S3 Fig) in HepG2 cells. These two inhibitors alone had no significant effect on HepG2 cell proliferation or apoptosis (Fig 3B and 3C, and S3 Fig). Liposomal C8 was significantly more potent than free C8 in inducing HepG2 cell apoptosis (S3 Fig).

To exclude possible off-target effect of the pharmacological inhibitors, we established a stable HepG2 cell line expressing the dominant negative JNK1 (dn-JNK1) (Fig 3D). As demonstrated, liposomal C8-induced JNK activation (Fig 3D), c-Jun expression (Fig 3D), and following cytotoxicity (Fig 3E and 3F) were largely inhibited in JNK1-mutated HepG2 cells.

Further studies were also performed to investigate the role of ASK1 in liposomal C8-induced actions. ShRNA strategy was applied. As demonstrated, targeted-shRNA significantly downregulated ASK1 expression in the stable HepG2 cells (Fig 3G). Importantly, liposomal C8-induced JNK activation and c-Jun expression were almost blocked in ASK1-silenced cells (Fig 3G). As a result, the anti-proliferative and pro-apoptotic activities by liposomal C8 were attenuated (Fig 3H and 3I). Notably, ASK1-JNK activation was also observed in liposomal C8-treatd SMMC-7721 and Huh-7 cells (Data not shown), and liposomal C8-induced cytotoxicity in above HCC cells was inhibited with SP600125 pre-treatment (Data not shown). Together, these results indicate that ASK1-JNK activation contributes to liposomal C8-induced *in vitro* anti-HCC activity.

### 3.4. Liposomal C8 ceramide inhibits AKT-mTOR activation in HCC cells

**Fig 4:**
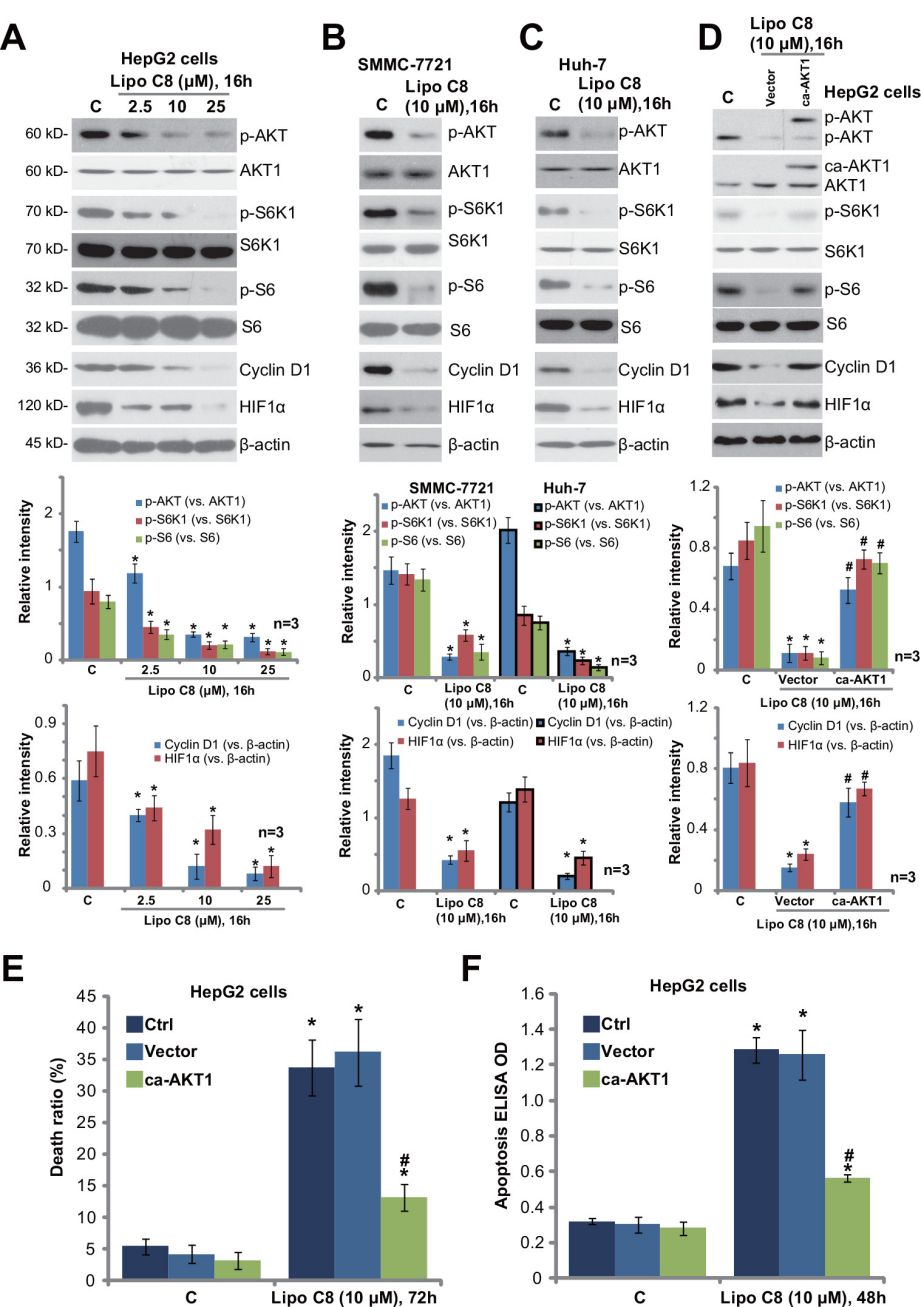
Liposomal C8 ceramide inhibits AKT-mTOR activation in HCC cells. HepG2 (A), SMMC-7721 (B) and Huh-7 (C) HCC cells were treated with applied concentrations of liposomal C8 for indicated time, expressions of indicated proteins were tested Western blots (A-C). Control HepG2 cells (“Ctrl”), as well as stable HepG2 cells expressing constitutively-active AKT1 (“ca-AKT1”) or empty vector (Ad-GFP) were treated with liposomal C8, Western blots were applied to test listed proteins (D), subsequent cell death (E) and apoptosis (F) were also tested. Expressions of indicated proteins were quantified (A-D, a total of three repeats). Data represent the means of three independent experiments ± SD. The asterisk (*) indicates statistically significant differences compared to “C” group. ^#^ indicates statistically significant differences compared to liposomal C8 only group of “Ctrl” cells (E and F).

Over-activation of AKT-mTOR (mammalian target of rapamycin) signaling plays an important role in regulating several key cancerous behaviors of HCC [22]. Existing evidences have demonstrated that free C6 and liposomal C6 could inhibit AKT-mTOR activation in HCC [15] and other cancer cells [23]. Thus, we tested AKT-mTOR activation in liposomal C8-treated HCC cells. Western blot results in Fig 4A demonstrated that liposomal C8 dramatically inhibited AKT and mTOR activation in HepG2 cells. Note that AKT activation was tested by phosphorylation at Ser-473, and mTOR activation was evidenced by phosphorylations of S6K1 (Thr-389) and S6 (Ser-235/236) [22]. The effect of liposomal C8 was again dose-dependent (Fig 4A). Meanwhile, liposomal C8 inhibited AKT-mTOR activation in two other HCC cell lines: SMMC-7721 (Fig 4B) and Huh-7 (Fig 4C). Further studies showed that expression of AKT-mTOR-regulated proteins, including cyclin D1 [24] and HIF1α [25,26], was both downregulated in liposomal C8-treated HCC cells (Fig 4A–4C). Notably, the liposomal vehicle control had no significant effect on AKT-mTOR activation in HCC cells (Data not shown).

To study the role of AKT-mTOR inactivation in liposomal C8’s actions, we introduced a constitutively-active AKT1 (“ca-AKT1”) into HepG2 cells, and stale cell line was selected (Fig 4D). Western blot results demonstrated that ca-AKT1 restored AKT-mTOR activation in liposomal C8-treated HepG2 cells (Fig 4D). Further, downregulation of cyclin D1 and HIF1α by liposomal C8 was again largely attenuated with ca-AKT1 introduction (Fig 4D). More importantly, following HepG2 cell death (Fig 4E) and apoptosis (Fig 4F) were significantly inhibited in ca-AKT1-expressing cells. These results indicate that AKT-mTOR inhibition also contributes to liposomal C8-induced actions in HCC cells.

### 3.5. Liposomal C8 ceramide inhibits HepG2 cell growth in SCID mice, while dramatically improving mice survival

**Fig 5:**
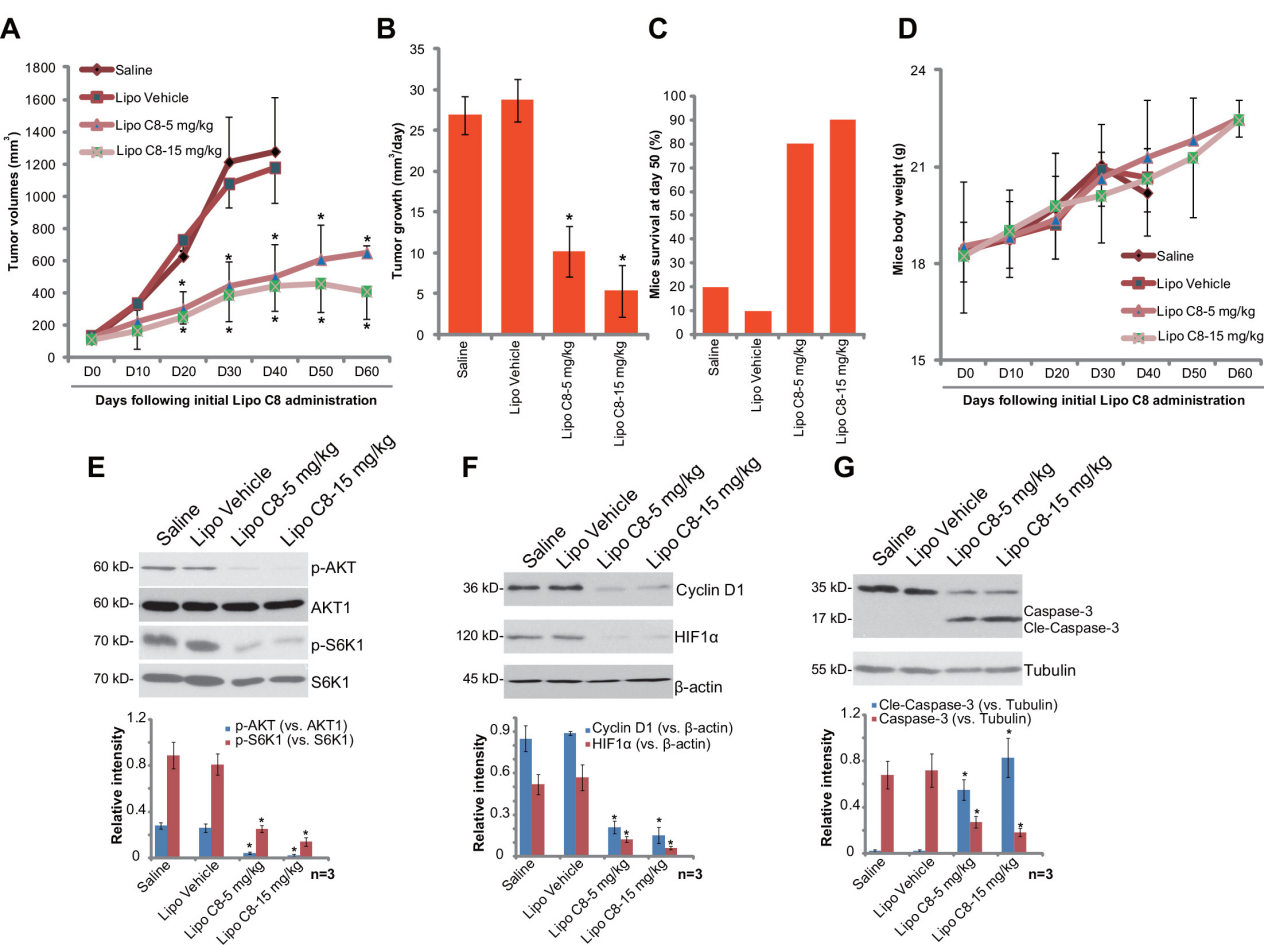
Liposomal C8 ceramide administration inhibits HepG2 cell growth in SCID mice, while dramatically improving mice survival. HepG2 xenograft-bearing SCID mice (10 mice per group) were intravenously (*i*.*v*.) injected with liposomal C8 (5/15 mg/kg body weight, once every two days, 30 days), liposomal vehicle control (no ceramide, “Lipo Ctrl”) or Saline, HepG2 xenograft volumes (A) and mice body weights (D) were recorded every 10 days, tumor growth (in mm^3^/day) was also presented (B). Mice survival at day 50 was shown (C). At day 50, xenografted tumors were isolated (n = 3 for each group), expression of listed proteins in the tumor tissues was tested by Western blot (E-G). Protein expression was quantified (E-G). *In vivo* experiments were repeated twice, and similar results were obtained. * indicates statistically significant differences compared to “Saline” group.

At last, we tested the *in vivo* anti-tumor activity of liposomal C8 [27]. HepG2-bearing SCID mice model was applied. The results demonstrated that *i*.*v*. injection of liposomal C8 at 5 and 15 mg/kg (body weight) dramatically inhibited HepG2 xenograft growth in SCID mice (Fig 5A and 5B). Liposomal vehicle (“Lipo Vehicle”) control showed no effect on xenograft growth. SCID mice survival was significantly improved following liposomal C8 administration (Fig 5C). The fast majority saline- or “Lipo Vehicle”-treated mice were already dead at day-50, as compared to most mice were still survival with liposomal C8 administration (Fig 5C). Note that mice body weights were almost not affected by the liposomal C8 regimens (Fig 5D), nor did we observe any signs of apparent toxicities (Data not shown), indicating overall safety of the liposomal C8 regimens [27]. Collectively, these results demonstrate that liposomal C8 administration inhibits HepG2 xenograft growth and improves SCID mice survival.

We tested the signaling change in xenograft tumors with or without liposomal C8 administration. Results in Fig 5E and 5F demonstrated clearly that liposomal C8 administration significantly inhibited phosphorylation of AKT and S6K1, whiling downregulating Cyclin D1 and HIF1α in xenografted tumor tissues. These signaling results are in line with the *in vitro* findings. We also examined cleaved-caspase-3 expression in the xenograft tumors. Results demonstrated that liposomal C8 induced significant caspase-3 cleavage in xenografted tumors, indicating apoptosis activation.

## Discussions

In the current study, we demonstrated that liposomal C8 inhibited HCC cell survival and proliferation, more potently than regular free C8 ceramide. Meanwhile, liposomal C8 activated caspase-dependent apoptosis in HCC cells, and liposomal C8-induced HCC cytotoxicity was attenuated by the caspase inhibitors. Further studies showed that liposomal C8 activated ASK1-JNK signaling, while inhibiting AKT-mTOR activation in HCC cells, which mediated its cytotoxicity. *In vivo*, liposomal C8 administration significantly inhibited HepG2 xenograft growth, and dramatically improved mice survival.

Here we showed that liposomal C8 induced ASK1-JNK activation and AKT-mTOR inactivation in HCC cells, both appeared important for subsequent HCC cell apoptosis. Pharmacologically and/or genetically reversing above signaling changes alleviated HCC cytotoxicity by liposomal C8. Yet, it appeared that JNK activation and AKT-mTOR inactivation were independent of each other. JNK inhibition, mutation or shRNA silence failed to restore AKT-mTOR activation in liposomal C8-treated cells (Data not shown). Further, ca-AKT1 introduction had no detectable effect on JNK activation by liposomal C8 (Data not shown). It should be noted that ASK1-JNK inhibition or ca-AKT1 introduction only inhibited, but not reversed, liposomal C8-induced cytotoxicity against HCC cells, indicating that other signalings could also play a role in mediating liposomal C8’s actions in HCC cells. For example, a recent study by Chen et al., showed that C6 ceramide activated AMP-activated protein kinase (AMPK)-dependent p53 signaling to promote cancer cell apoptosis [27]. It was also shown that C6 ceramide facilitated JNK-p53 activation to promote human colon cancer cell apoptosis [21]. Ji et al., demonstrated that endogenous ceramide production by perifosine activated pro-apoptotic JNK, p53 and AMPK signalings to mediate cell apoptosis [28]. Therefore, further studies will be needed to explore the role possible other signalings (i.e. AMPK and p53) in liposomal C8’s effects in HCC cells, and their link with ASK1-JNK and AKT-mTOR signalings.

Intriguingly, we showed that the cytotoxicity by liposome C8 ceramide was only observed in HCC cells, but not in non-cancerous HL7702 hepatocytes, suggesting a neoplasm-selective mechanism. *In vivo*, liposomal C8 injection also failed to induce apparent toxicities to tested mice. Our results here are consistent with other studies using other liposomal systems [29,30]. For example, Zolnik et al., showed that liposome short-chain ceramide could offer rapid tissue distribution without adverse effects [12]. One explanation could be the AKT-mTOR regulation by liposomal C8 in HCC cells. AKT-mTOR is often over-activated in HCC (see Fig 4) [22], contributing to cancer cell survival and apoptosis-resistance. Here we showed that liposomal C8 treatment significantly inhibited AKT-mTOR activation in HCC cells. While in non-cancerous HL7702 hepatocytes, basal AKT-mTOR is low and almost undetected (Data not shown), thus may not be a key determent of cell survival. The detailed signaling mechanisms of AKT-mTOR inactivation by liposomal C8 need further investigations.

## Conclusions

In summary, we demonstrate that liposomal C8 ceramide exerts potent anti-tumor activity in pre-clinical HCC models. Molecularly, ASK1-JNK activation and AKT-mTOR inactivation mediate, at least in part, liposomal C8-induced actions in HCC cells. Our *in vitro* findings along with *in vivo* results support further development of liposomal C8 as a novel and efficient anti-HCC agent.

## Supporting Information

S1 Fig SMMC-7721 and Huh-7 human HCC cells, as well as HL7702 human hepatocytes were treated with applied concentrations of free C8 ceramide for 72 h, cell survival was tested by MTT assay. Data represent the means of three independent experiments ± SD.
(EPS)

S2 Fig HepG2 cells were treated with applied concentrations of liposomal C8 for 48 h, representative TUNEL/DAPI staining images were presented. Bar = 25 μm. Data represent three independent experiments.
(EPS)

S3 Fig HepG2 cells, pretreated with or without JNK inhibitor IX (“JNKi”, 0.25 μM) or SP600125 (“SP”, 5 μM) for 1 h, were treated with liposomal C8 (10 μM) or free C8 (10 μM) for 48 h, cell apoptosis was analyzed by TUNEL staining assay (A) and Annexin V FACS assay (B). Data represent the means of three independent experiments ± SD. The asterisks (*) indicate statistically significant differences compared to “C” group. ^#^ indicates statistically significant differences compared to liposomal C8 only group.
(EPS)
